# Coping Style in Glioma Patients and Their Caregiver: Evaluation During Disease Trajectory

**DOI:** 10.3389/fneur.2021.709132

**Published:** 2021-09-01

**Authors:** Lara Guariglia, Sonia Ieraci, Veronica Villani, Antonio Tanzilli, Dario Benincasa, Francesca Sperati, Irene Terrenato, Andrea Pace

**Affiliations:** ^1^Psychology Unit, Istituto di Ricovero e Cura a Carattere Scientifico Regina Elena Cancer Institute, Rome, Italy; ^2^Neuro-Oncology Unit, Istituto di Ricovero e Cura a Carattere Scientifico Regina Elena Cancer Institute, Rome, Italy; ^3^Biostatistics and Bioinformatic Unit-Scientific Direction, Istituto di Ricovero e Cura a Carattere Scientifico San Gallicano Dermatological Insititute, Rome, Italy; ^4^Biostatistics and Bioinformatic Unit-Scientific Direction, Istituto di Ricovero e Cura a Carattere Scientifico Regina Elena National Cancer Institute, Rome, Italy

**Keywords:** coping style, glioma, dyadic model, distress, quality of life, palliative care

## Abstract

**Background:** Patients with glioma have a poor prognosis and, in a short period of time, have to deal with severe forms of disability, which compromise their psychological distress and quality of life. The caregivers of these patients consequently carry a heavy burden in terms of emotional and patient care. The study aims to evaluate the coping strategies of patients and their caregivers during the course of the disease in order to frame the adaptation process in a rapidly progressing pathology.

**Methods:** A prospective study on 24 dyads of patients affected by malignant glioma and their caregivers was conducted between May 2016 and July 2018. Questionnaires designed to identify the coping style (MINI-MaC Scale) and psychological distress (HADS scores) and assess QOL (EQ-5D) were administered at two time points: at first lines of treatment and at disease recurrence.

**Results:** Patients and their caregiver structure adaptive coping strategies during the disease: a coping style oriented toward a fighting spirit prevails at baseline (Mini-Mac Mean 3.23); fatalism prevails at recurrence (Mini-Mac Mean 3.03). Psychological distress affects the coping style expressed: high levels of anxiety symptoms were found to be significantly associated with a coping style oriented toward anxious preoccupation, helpless–hopeless, and fatalism; low depressive symptoms were inversely correlated with fighting spirit coping style. Patients' and caregivers' perceptions of quality of life were correlated between them and with performance status assessed by clinicians. In a dyadic perspective, the adaptation of a member of the couple varies as a function of the other partner's coping style.

**Conclusions:** Our data are in line with previous literature on cancer patients, demonstrating that coping style is not a persistent dimension of personality, but can change depending on the situation. Despite the disease rapid course, patients and their caregivers can structure adaptive and functional defenses to manage the disease.

## Introduction

Patients diagnosed with glioma have a poor prognosis and, despite increased treatment options, a limited survival (1–3). Fear about death, rapid physical decline, disease burden, difficult medical decisions, and desire for information about the disease induce, both patients and caregivers, to define adaptive strategies to deal with the disease burden. According to Lazarus' transactional approach to stress (4), coping can be defined as “constantly changing cognitive and behavioral efforts to manage specific external or internal demands that are appraised as taxing or exceeding the resources of a person.” Coping may be organized into five categories: fighting spirit, cognitive avoidance, anxious preoccupation, helpless–hopeless, and fatalism (4, 5). Coping strategies are considered a determinant factor in the process of emotional adaptation to the disease and may influence health-related quality of life (HRQoL) perception and psychological status in cancer patients (6–8). According to the theoretical model (4), coping strategies may change over time in different stages of the disease and are influenced by several factors, such as quality of life, cognitive function, different psychological distress features, clinical condition, and disease awareness (8–10). In addition, an analysis of coping strategies should take into account the dynamic interplay between partners, such as the dyad made by the patient and his/her main caregiver (11). The origin of the stress, the goals, the appraisals, and the coping strategies of each individual and patient/caregiver dyads need to be considered.

Several studies have evaluated coping styles in cancer patients, focusing both on individual and relational (dyadic) coping; however, brain tumor (BT) patients require a special approach due to the particular trajectory of the disease, the very poor prognosis, and the presence of cognitive and behavioral changes induced by the tumor in the brain. Malignant gliomas present a median survival of 17–36 months, and, despite aggressive treatments, the majority of patients will experience disease recurrence during the first years after diagnosis (EANO Guidelines) (12).

In this prospective, longitudinal study, we hypothesize that despite the short course and the aggressive nature of the disease, patients are still able to find adaptive strategies and change their coping style in relation to factors previously identified in the literature: quality of life, distress, and relational structure.

## Patients and Methods

A prospective study on 24 dyads of patients affected by newly diagnosed malignant glioma and their caregivers was conducted at IRCCS Regina Elena Cancer Institute in Rome between May 2016 and July 2018. The inclusion criteria were patients with newly diagnosed high-grade glioma, who were subjected to first-line treatment (surgery, radiotherapy, chemotherapy) without serious cognitive impairments that compromised the ability to understand and respond to questionnaires. All patients received a comprehensive clinical evaluation including psychological assessment, cognitive functions evaluation, and quality-of-life measurements. All caregivers were patients' relatives. Coping style, quality of life, and anxiety and mood were assessed at baseline, after diagnosis, and at the recurrence of the disease. All subjects provided written informed consent. The sociodemographic and clinical characteristics were collected using medical records. All patients included in this study were preliminarily assessed with the Italian version of the Mini-Mental State Examination (MMSE) (13, 14) and did not show relevant cognitive deficits. Patients' and caregivers' characteristics are shown in Table 1.

**Table 1:** Patients' and caregivers' characteristics.

**Characteristics**	**Patients, *n* (%)**	**Caregivers, *n* (%)**
Histology:	Glioblastoma 22 (92)	
	Anaplastic astrocytoma 2 (8)	
Evaluated at baseline	24	24
Evaluated at recurrence	8	8
Age in years, median (min–max)	58 (31–76)	
Males/females	14/10 (58/42)	7/17(29/71)
Patients educational level	Elementary 3 (12)	
	Lower secondary 5 (21)	
	Upper secondary 9 (38)	
	Graduate 7 (29)	
Baseline Karnofsky, median (min–max)	90 (70–100)	
Karnofsky at follow-up, median (min–max)	70 (60–100)	
baseline MMSE, median (min–max)	30 (25–30)	
MMSE at follow-up, median (min–max)	28 (24–30)	

### Assessment Tools

Styles of coping of patients and caregivers were evaluated using the Mini-Mental Adjustment to Cancer (Mini-MAC) scale (15).

The Mini-MAC is a revised version of the widely used Mental Adjustment to Cancer scale (15), developed for measuring mental adjustment to cancer in a general cancer population. The Mini-MAC has five domains: Fighting Spirit (FS; four items); Helpless–Hopeless (HH; eight items); Anxious Preoccupation (AP; eight items); Fatalism (FA; five items); and Cognitive Avoidance, (CA; four items). It is composed of 29 questions relating to the five coping strategies. The items are rated on a four-point Likert scale ranging from “Definitely does not apply to me” (1) to “Definitely applies to me” (4) and measures the patients' experiences at present. A higher score represents a higher endorsement of the adjustment response. The domains can be scored separately through simple addition. Since the domains consist of a different number of items, we also calculated mean scores by dividing the sum by the number of items.

The Hospital Anxiety and Depression scale was used to evaluate the level of distress both in patients and in caregivers (7, 16). The questionnaire comprises seven questions for anxiety and seven questions for depression. For both scales, scores <7 indicate absent anxiety/depression; scores between 8 and 10 indicate a mild level of anxiety and depression; scores between 11 and 14 indicate a moderate level of anxiety and depression; and scores between 15 and 21 indicate severe-level anxiety and depression.

Patients' quality of life was assessed using the Italian version of the EuroQol-5D (EQ-5D) questionnaire, obtaining a patient's self-evaluation and a caregiver's evaluation of the patient's health status (17). The questionnaire has two components: health state description and evaluation. In the description part, health status is measured in terms of five dimensions: mobility, self-care, usual activities, pain/discomfort, and anxiety/depression. In the evaluation part, the respondents evaluate their overall health status using the visual analog scale (EQ-VAS). Patients' performance status was assessed by clinicians using the Karnofsky scale.

Questionnaires and interviews were handed out on paper by two psychologists (LG and SI).

### Statistics

Descriptive statistics were calculated for all variables of interest. Continuous variables were reported through means and their relative standard deviations, while categorical variables were synthetized with frequencies and percentage values. All continuous variables were tested for normality. The non-parametric Spearman's rank correlation coefficient was used to evaluate the correlation between the different categories of copying. Statistical significance was considered when *p*-value ≤ 0.05. All analyses were carried out with SPSS v 21.0.

## Results

Between May 2016 and July 2018, 24 patients affected by malignant glioma and their respective main caregivers were assessed during the first cycle of adjuvant chemotherapy (baseline). Patients and caregivers were recruited at Neuro-Oncology Department of Regina Elena Cancer Institute in Rome, Italy. The first evaluation was 4.3 months after diagnosis, on average (range 1.6–6.7 months). Eight patients and their respective caregivers were reassessed after a recurrence of glioma, on average 12.1 months after diagnosis. At recurrence, 16 patients were not evaluable due to disease progression with severe neurocognitive impairment (10 patients) or lost at follow-up (6 patients).

The average interval between the first and second evaluations was 7.3 months (range 4–13 months). Main caregivers were spouses (*n* = 18), sons (*n* = 4), or parents (*n* = 2). Patients' and caregivers' characteristics are demonstrated in Table 1.

### Patients' Coping Styles

#### Baseline

At baseline, at group level, patients reported higher scores in the domain of FS (mean 3.23; SD 0.82) and CA (mean 3.05; SD 0.51). The domains of FA (FA mean 2.77, SD 0.82) and AP (AP mean 2.29, SD 0.71) reached a slightly lower average score. Detailed Mean Score values are described in Table 2.

**Table 2:** Results of assessments at baseline and recurrence in patients and caregivers.

	**Baseline (** ***n*** **=** **24)**		**Recurrence (** ***n*** **=** **8)**	
	**Mean (SD)**	**Min–max**	**Mean (SD)**	**Min–max**
**Patients**				
FS	3.23 (0.82)	1.75–4.00	2.90 (0.68)	1.75–3.75
HH	1.84 (0.57)	1.00–3.38	2.06 (0.42)	1.50–2.75
AP	2.29 (0.71)	1.13–3.50	2.47 (0.61)	1.88–3.38
FA	2.77 (0.82)	1.40–4.00	3.03 (0.64)	2.40–4.00
CA	3.05 (0.51)	2–4	2.53 (0.66)	1.75–3.50
**Caregivers**				
FS	2.93 (0.64)	1.50–4.00	2.90 (0.42)	2.50–3.75
HH	1.83 (0.55)	1.00–3.38	2.00 (0.53)	1.25–2.88
AP	2.90 (0.62)	1.75–4.00	2.89 (0.44)	2.25–3.63
FA	2.65 (0.58)	1.40–3.60	2.85 (0.30)	2.40–3.20
CA	2.84 (0.70)	1.25–4.00	2.56 (0.40)	2.00–3.00

*FS, fighting spirit; HH, helpless–hopeless AP, anxious preoccupation; FA, fatalism; CA, cognitive avoidance*.

**Figure 1:**
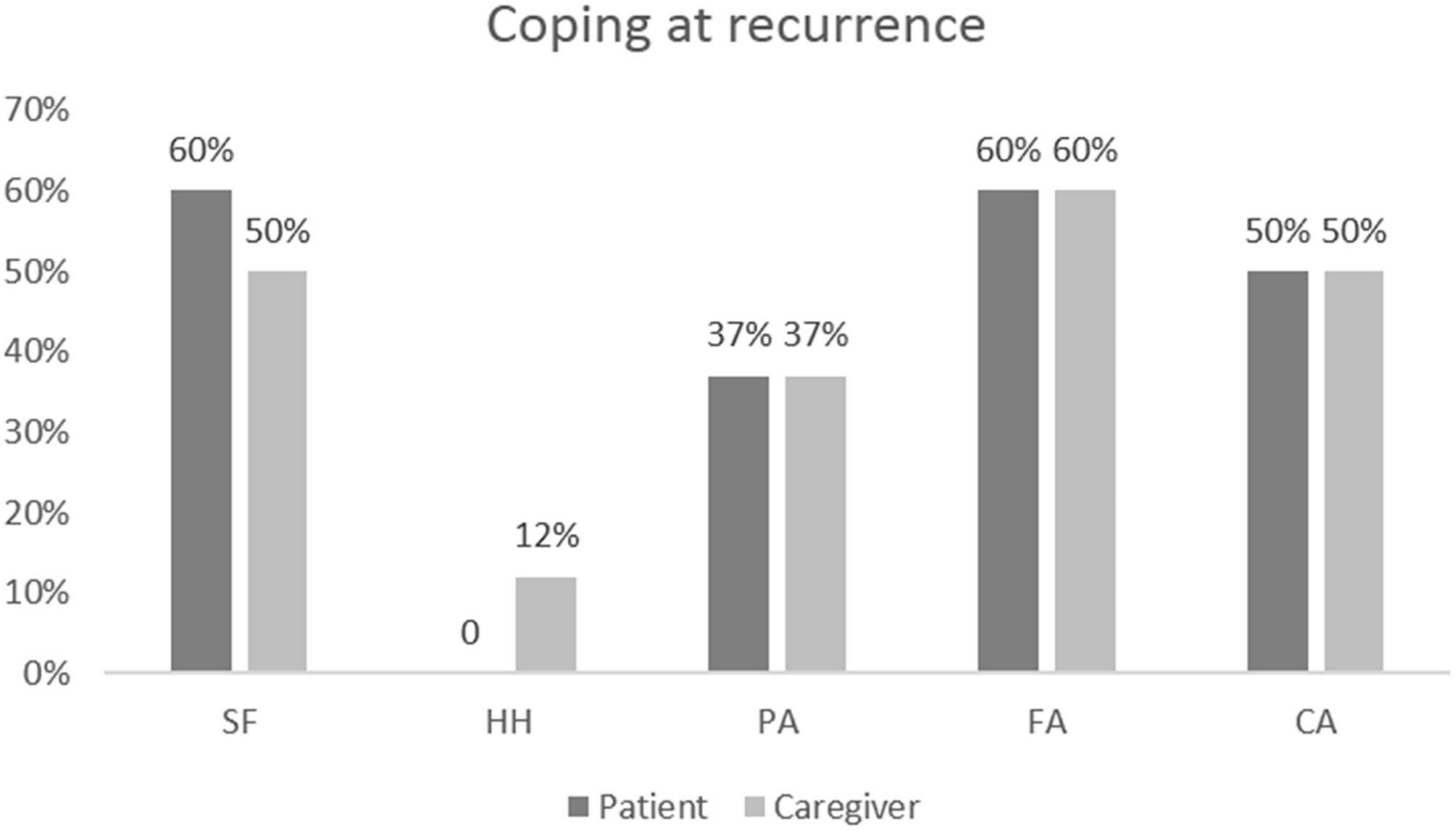
Results of coping assessments at baseline in patients and caregivers at the individual level.

At the individual level, most frequent coping strategies resulted in CA (18 patients, 75%) and FS (17 patients, 70%). However, 33% of responders displayed a predominant coping style in the domain of FA and 25% in the domain of AP (Figure 1).

High levels of anxiety symptoms measured with the Hospital Anxiety and Depression (HAD) scale at baseline were found to be significantly associated with a coping style oriented toward anxious preoccupation (baseline: Rho = 0.618, *p* = 0.001), while low depressive symptoms were inversely correlated with fighting spirit coping style, although this finding did not reach a full statistical significance (Rho = −0.398, *p* = 0.054).

Patients' self-perception of a high quality of life, measured with EQ5 VAS, was directly correlated with FA (Rho = 0.727, *p* = 0.041). There was no statistically significant correlation between age, sex, educational level, and adopted coping style; in the same way, no statistically significant correlations were observed between the functional status measured by the Karnovsky scale and the coping styles adopted.

#### Recurrence

A longitudinal evaluation was possible only in eight patients and their caregivers due to early disease progression, cognitive deficits, or patients lost at follow-up. At recurrence, at a group level, patients evaluated reported higher scores in the domain of FA (mean 3.03; SD 0.64) and FS (mean 2.90; SD 0.68). The domains of CA (mean 2.53; SD 0.66) and AP (mean 2.47; SD 0.61) reach a slightly lower average score (Table 2).

**Figure 2:**
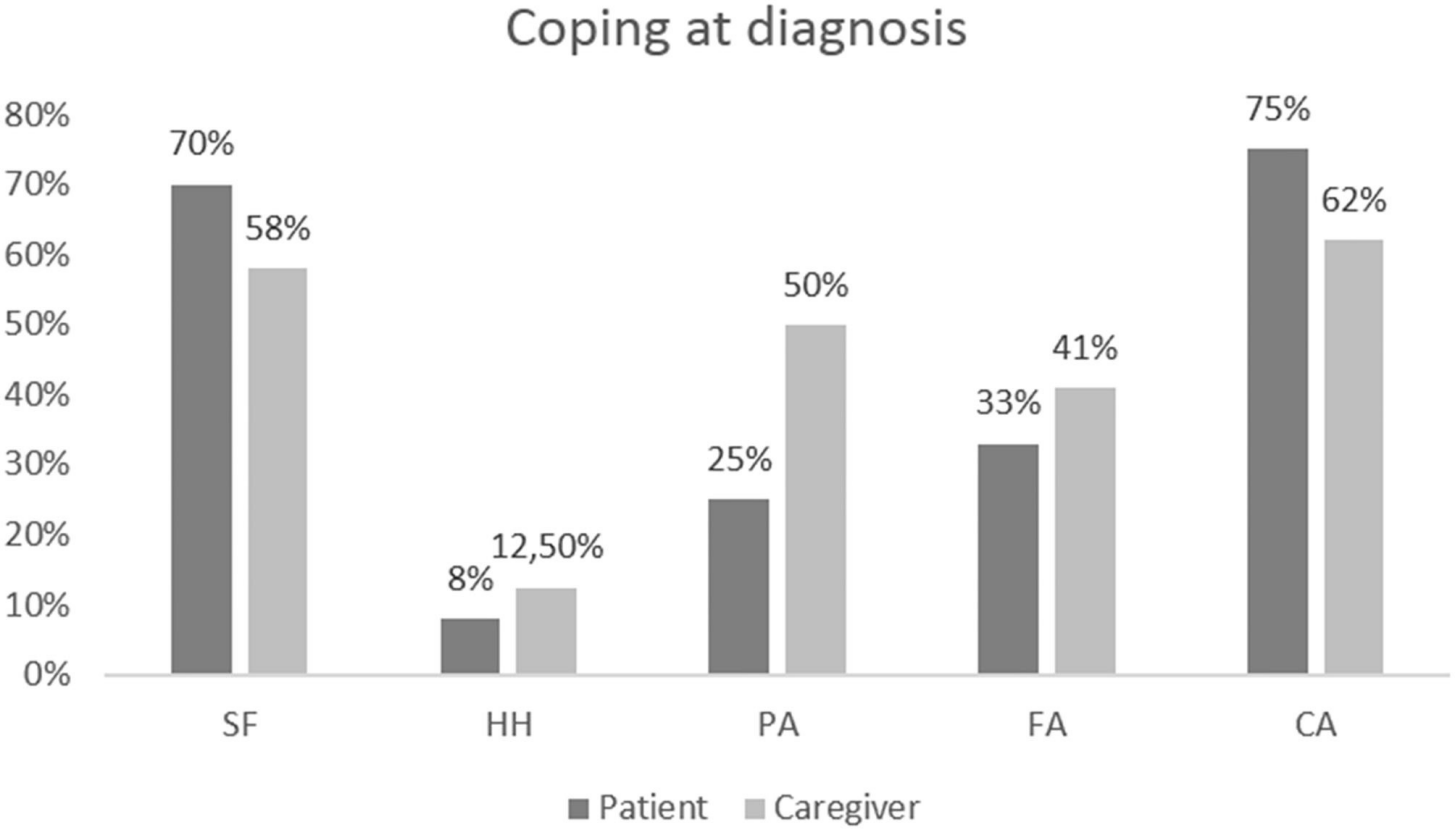
Results of coping assessments at recurrence in patients and caregivers at the individual level.

At an individual level, patients evaluated showed a higher score in the domain of FS (60%) and FA (60%). Avoidance coping style was present in 50% of patients (Figure 2).

At recurrence, a high score of anxiety symptoms was associated with a coping style oriented toward the domains of HH (Rho = 0.789, *p* = 0.020), AP (Rho = 0.895, *p* < 0.003), and FA (Rho 0.821, *p* = 0.012). The presence of depressive symptoms was found to be associated with a coping strategy predominantly in the domain of HH (Rho = 0.867, *p* = 0.005) and AP (Rho = 0.957, *p* < 0.001) and inversely correlated with FS (Rho = −0.845, *p* = 0.008). At recurrence, patients' self-perception of low quality of life was correlated with anxious preoccupation coping style (Rho = −0.780, *p* = 0.022).

### Caregivers' Coping Styles

#### Baseline

At baseline, at the group level, caregiver coping style analysis showed a higher score in the domain of FS (mean 2.93; SD 0.64) and AP (mean 2.90; SD 0.62). The domains of CA (mean 2.84; SD 0.70) and FA (mean 2.65; SD 0.58) reached a slightly lower average score. Detailed mean score values are described in Table 2. At the individual level, 54% of caregivers presented a coping style predominantly oriented toward the domain of fighting spirit and avoidance, and 50% showed a high score in the domain of anxious preoccupation. HAD score measures in caregivers at baseline showed that low levels of anxiety and depression were associated with a coping style oriented toward a fighting spirit (Rho = −0.586, *p* = 0.003 for anxiety and Rho = −0.691, *p* < 0.001 for depression); high levels of anxiety were associated with a coping style oriented toward anxious preoccupation (Rho = 0.456, *p* = 0.025); and high levels of depression were associated with AP (Rho = 0.480, *p* = 0.018) and HH (Rho = 0.581, *p* = 0.003). Perception by the caregiver of a low patient quality of life at baseline was correlated with HH (Rho = 0.484, *p* = 0.016) and with AP (Rho = 0.619, *p* = 0.001). On the contrary, perception of a better patient quality of life was correlated with FS (Rho = 0.747, *p* = 0.033) and inversely correlated with HH (Rho = −0.926, *p* = 0.001).

#### Recurrence

At recurrence, caregiver coping style analysis showed a higher score in the domains of FS (mean 2.90, SD 0.42) and AP (mean 2.89, SD 0.44). The domains of FA (mean 2.85, SD 0.30) and CA (mean 2.56, SD 0.40) reached a fairly high average score. Detailed mean score values are described in Table 2.

At the individual level, 62% of caregivers presented a coping style predominantly oriented toward the domain of FS and FA; 50% presented a coping style oriented toward AP; 37% toward CA (Figure 2).

At recurrence, low levels of anxiety were associated with an FS coping style (Rho = −0.907, *p* = 0.002).

### Dyadic Coping

Analyzing the interconnection between patient and caregiver dyad, at baseline, 58% of couples show complementary coping styles: patients with HH have caregivers with AP (Rho 0.431, *p* = 0.036); patients with FA have a caregiver with an FS (Rho = 0.462, *p* = 0.023); and patients with FS have a caregiver with HH (Rho = −0.434, *p* = 0.034).

However, 42% showed symmetrical coping: an FS and a HH coping in the patient was significantly correlated with the same style of coping in the caregiver (Rho = 0.539, *p* = 0.007 and Rho = 0.448, *p* = 0.028, respectively). At recurrence, among the eight dyadic couples examined, 80% showed symmetrical coping: HH coping in the patient was significantly correlated with the same coping style in the caregiver (Rho = 0.813, *p* = 0.014). However, 20% of dyadic couples showed a complementary coping: patients with AP have caregivers with HH (Rho = 0.809, *p* = 0.015). An inverse weak correlation was found between avoidance style in the patient and in the caregiver (Rho = −0.716, *p* = 0.046), which means that when a member of the couple uses avoidant coping, the other is unable to use the same strategy (Figure 1).

Concerning the quality-of-life evaluation, our results show that at baseline, patients' and caregivers' perceptions of patients' quality of life were correlated (Rho = 0.725, *p* < 0.0001). Also at recurrence, the patient health status self-assessment was correlated with the caregiver evaluation (EQ-VAS: Rho = 0.753, *p* = 0.031). Quality of life, as assessed both by patient and by caregiver, was correlated with performance status assessed by clinicians (Rho = −0.546, *p* < 0.006; Rho = −0.642, *p* < 0.001).

## Discussion

Brain tumors represent a devastating disease and the poor prognosis, and the short history of disease renders this tumor quite different with respect to other cancers. Our prospective, longitudinal study is aimed to evaluate how BT patients and their caregivers organize the response to stress utilizing strategies to manage the disease and related symptoms.

Our results show that, both at baseline and recurrence, patients' coping strategies are not strongly polarized but showed many different styles facing the new situation. However, most BT patients initially face the disease either with a fighting spirit or by a defensive cognitive avoidance style; after recurrence, many patients maintained a fighting spirit but the cognitive avoidance coping style boils down to fatalism.

Despite the aggressiveness of the disease and the poor prognosis, during first-line treatment, most of the patients can display functional coping strategies, such as FS, which favors active participation and adherence to treatment; in addition, CA style preserves the individual from excessive exposure to distress. On the other hand, HH, which is considered the most dysfunctional style of coping, is the least expressed at baseline evaluation. The functional coping strategies are also preserved at disease recurrence but with a progressive adherence to the reality: CA is replaced by FA.

The caregivers' coping strategy initially face the patient's disease with an FS or by an AP style and seem to maintain the same adaptation strategy at disease recurrence.

Concerning the correlation between coping style and anxious/depression and HRQOL, our data show that, both at baseline and recurrence, in patients and caregivers, high levels of anxiety/depression and low perception of HRQoL were significantly associated with a higher score on the HH and AP domains. In addition, higher levels of anxiety and depression observed in caregivers at baseline were correlated with a higher score in the domain of AP.

These data confirm previous evidence of a strong association of anxious and depressive symptoms with coping strategies (9, 10, 18). Similarly, our data confirm previous observations on cancer patients, showing that perceptions of HRQoL correlate with coping strategies (9, 10, 18). However, probably due to the small sample size, in our study, a significant correlation between HRQoL and coping style was observed only between EQ5 VAS score, a visual analogical scale and, therefore, with a greater degree of approximation, and coping style oriented toward AP and HH domains, mainly in caregivers' perception.

Our data are in line with previous literature on cancer patients, demonstrating that coping style is not a persistent dimension of personality, but can change depending on the situation. In addition, patients' and caregivers' reactions could be different, although a mutual influence was present, according to a dyadic model.

Few studies have examined the patient/caregiver interaction model in BT. Other studies in cancer patients have reported that the relation of one partner's coping to adjustment varies as a function of the other partner's coping style (19).

The theoretical model of dyadic coping describes coping change not only based on each one's resources but also on the couple relationship that engendered a mutual influence and consistency (19). When coping is considered in a dyadic perspective, in some cases patients and caregivers assume a symmetrical attitude and provide the same coping response to the disease, establishing a supportive relationship; in other cases, patients and caregivers assume a complementary attitude: one of the two members assumes a style of coping that is contrary to the other, establishing a compensative relationship (19).

In the early period of the disease, one subject takes charge of facing reality letting the other keep in a defense attitude (i.e., when the disease is faced with AP by the caregiver and with an FS by the patient).

At recurrence, the couple most frequently maintains similar coping strategies and reinforce each other (i.e., when the disease is faced with FA or CA from both). Our data show a consistent difference between the baseline assessment and that at disease recurrence: at baseline, 42% of couples express a symmetrical relationship between the coping styles, while at the recurrence of the disease, the percentage achieves 80%. Therefore, during the course of the disease, couples progressively settle on the expression of the same coping style.

Although the results of the longitudinal assessment are limited by the small number of patients/caregivers receiving a follow-up evaluation, our results show that, after a few months since baseline assessment, there is a modification of coping strategies observed at disease recurrence with a shift toward AP and FA, probably related to a higher score of anxious/depression and perception of lower quality of life. This aspect represents probably the main difference between BT and other cancer patients due to the rapid deterioration of clinical conditions and a short time to recurrence in neuro-oncological patients.

The results of our study provide important insights into coping strategies adopted by patients with malignant gliomas and their caregivers along the disease trajectory.

Considering the short life expectancy of malignant glioma patients and their care needs throughout the disease trajectory, coping strategies should be considered as a key component in the management of BT patients. Patients' coping styles have an important influence in critical aspects of care such as communication of diagnosis and prognosis, discussion with patients and their caregivers about the goal of treatments, early introduction of palliative care, and advanced planning of patients' preferences concerning the end-of-life treatment and issues.

Despite the well-recognized importance to improve patient–clinician communication about illness and prognosis and early integration of palliative care in the trajectory of disease of BT patients, recent studies on prognostic awareness and preferences for prognostic communication in BT patients reported that some BT patients wish that prognosis was discussed in greater depth and earlier in the disease course, but others do not want to discuss prognosis fully, especially when the discussion is experienced as deleterious to maintaining hope (20). In addition, in a qualitative study on preferences for information about prognosis, comprehension of information, and satisfaction with information, 50% of participants preferred to receive “all information” while the remainder wanted only “important” or “critical” information (21).

According to recent studies in advanced cancer, the coping style adopted may strongly influence patients' prognostic awareness and patients' availability to participate in prognosis discussions (10).

Moreover, patients' prognosis awareness fluctuates longitudinally through disease and treatment courses. Considering the strong interaction between patients' coping strategies and critical issues related to communication and early integration of palliative care, additional studies on timing and ways of discussing prognosis and goals of care in this population are needed.

The most important limits of this study are the small sample size and the small number of dyadic couples receiving a longitudinal assessment. Moreover, the inclusion in this study of patients without cognitive deficits may lead to a selection bias with the exclusion of patients with lower performance status.

## Data Availability Statement

The raw data supporting the conclusions of this article will be made available by the authors, without undue reservation.

## Ethics Statement

Ethical review and approval was not required for the study on human participants in accordance with the local legislation and institutional requirements. The patients/participants provided their written informed consent to participate in this study.

## Author Contributions

LG and AP contributed to the conception and design of the study. LG, SI, VV, AT, and DB administered the questionnaires and assessed patients. FS and IT performed the statistical analysis. All authors contributed to the manuscript revision, read, and approved the submitted version.

## Conflict of Interest

The authors declare that the research was conducted in the absence of any commercial or financial relationships that could be construed as a potential conflict of interest.

## Publisher's Note

All claims expressed in this article are solely those of the authors and do not necessarily represent those of their affiliated organizations, or those of the publisher, the editors and the reviewers. Any product that may be evaluated in this article, or claim that may be made by its manufacturer, is not guaranteed or endorsed by the publisher.
